# Cytotoxic effect of 5-aminoimidazole-4-carboxamide-1-β-4-ribofuranoside (AICAR) on childhood acute lymphoblastic leukemia (ALL) cells: implication for targeted therapy

**DOI:** 10.1186/1476-4598-6-46

**Published:** 2007-07-10

**Authors:** Tapas K Sengupta, Gilles M Leclerc, Ting Ting Hsieh-Kinser, Guy J Leclerc, Inderjit Singh, Julio C Barredo

**Affiliations:** 1 Department of Pediatrics, University of Miami Miller School of Medicine, Miami, FL 33101, USA; 2 Department of Pediatrics, Medical University of South Carolina, Charleston, SC 29425, USA; 3 School of Life Sciences, Indian Institute of Science Education and Research, Kolkata 700106, India

## Abstract

**Background:**

Acute lymphoblastic leukemia (ALL) is the most common hematological malignancy affecting children. Despite significant progress and success in the treatment of ALL, a significant number of children continue to relapse and for them, outcome remains poor. Therefore, the search for novel therapeutic approaches is warranted. The aim of this study was to investigate the AMP activated protein kinase (AMPK) as a potential target in childhood acute lymphoblastic leukemia (ALL) subtypes characterized by non-random translocation signature profiles. We evaluated the effects of the AMPK activator AICAR on cell growth, cell cycle regulators and apoptosis of various childhood ALL cells.

**Results:**

We found that treatment with AICAR inhibited cell proliferation, induced cell cycle arrest in G1-phase, and apoptosis in CCRF-CEM (T-ALL), NALM6 (Bp-ALL), REH (Bp-ALL, TEL/AML1) and SupB15 (Bp-ALL, BCR/ABL) cells. These effects were abolished by treatment with the adenosine kinase inhibitor 5'-iodotubericidin prior to addition of AICAR indicating that AICAR's cytotoxicity is mediated through AMPK activation. Moreover, we determined that growth inhibition exerted by AICAR was associated with activation of p38-MAPK and increased expression of the cell cycle regulators p27 and p53. We also demonstrated that AICAR mediated apoptosis through the mitochondrial pathway as revealed by the release of cytochrome C and cleavage of caspase 9. Additionally, AICAR treatment resulted in phosphorylation of Akt suggesting that activation of the PI3K/Akt pathway may represent a compensatory survival mechanism in response to apoptosis and/or cell cycle arrest. Combined treatment with AICAR and the mTOR inhibitor rapamycin resulted in additive anti-proliferative activity ALL cells.

**Conclusion:**

AICAR-mediated AMPK activation was found to be a proficient cytotoxic agent in ALL cells and the mechanism of its anti-proliferative and apoptotic effect appear to be mediated via activation of p38-MAPK pathway, increased expression of cell cycle inhibitory proteins p27 and p53, and downstream effects on the mTOR pathway, hence exhibiting therapeutic potential as a molecular target for the treatment of childhood ALL. Therefore, activation of AMPK by AICAR represents a novel approach to targeted therapy, and suggests a role for AICAR in combination therapy with inhibitors of the PI3K/Akt/mTOR pathways for the treatment of childhood in ALL.

## Background

AMP activated protein kinase (AMPK) is a highly conserved heterotrimeric serine/threonine protein kinase that regulates the intracellular ratio of AMP to ATP, and it is activated under conditions that deplete cellular ATP and hence increase AMP levels [1-3]. Therefore, the AMPK cascade is a sensor of cellular energy status that is activated by multiple stimuli such as metabolic stresses including ischemia, hypoxia and glucose deprivation, environmental stresses like heat shock, oxidative and osmotic stress [4,5]. It is also activated by various pharmacological agents including respiratory chain inhibitors (actinomycin D, nitric oxide), ATP synthase inhibitors (oligomycin), mitochondrial uncouplers (dinitrophenol), TCA cycle inhibitors (arsenite), biguanides (metformin) and nucleosides (adenosine analogue AICAR) [6-9]. The AMPK pathway is also implicated in the regulation of cell cycle and cell proliferation and it has recently been determined that its activation by AICAR results in pro-apoptotic effect [10-12].

Acute lymphoblastic leukemia (ALL) is the most common hematological malignancy affecting children and adolescents [13]. Significant advances in our understanding of the biology and molecular genetics of ALL have led to the identification of molecularly defined subgroups important for therapy stratification and prognosis [14]. Despite significant progress and success in the treatment of ALL, a significant number of children continue to relapse and for them, outcome remains poor [14]. Likewise, the outcome for others who are diagnosed with chemotherapy resistant phenotypes continues to be poor. In this context, childhood ALL continues to be a major cause of cancer related mortality in children and adolescents and therefore, novel treatment strategies are needed. During recent years, novel targeted and molecular agents have been introduced in the treatment of hematological malignancies in adults [15], but the experience with these agents in pediatric leukemia remains minimal. Our data presented herein, supports the role of AMPK and its downstream pathways as a suitable target for molecular therapies in childhood ALL. The recognition of this pathway's physiological importance in terms of cell cycle regulation, cell proliferation, survival and apoptosis is highlighted by recent reports in prostatic and breast carcinomas, as well as gliomas, among others [16,17].

The anti-proliferative and pro-apoptotic activity of AMPK have been linked to the tumor suppressor genes LKB1 (a serine/threonine protein kinase formerly identified as STK11) and TSC2 tuberous sclerosis complex 2) [6,18,19]. LKB1 mutations result in Peutz-Jeghers syndrome, which leads to predisposition to cancers of the colon, pancreas, breast, and other sites [20-22]. Mutations of LKB1 typically occur in the catalytic domain, leading to loss of its kinase activity [23]. TSC2 forms a complex with TSC1 and inhibits mTOR mammalian target of rapamycin), leading to inhibition in protein synthesis and negative regulation of cell size and growth [24]. Mutations of TSC1·TSC2 cause tuberous sclerosis, a condition associated with hamartomatous polyps in multiple tissues and an increased risk of cancers [25].

Structurally, AMPK consists of a catalytic (α) and two regulatory subunits (β and γ), each subunit having at least two isoforms [1,26]. AMPK activation requires a conformational change induced by AMP binding to the α and γ subunits, which in turn allows its phosphorylation/activation by the upstream protein kinase LBK1 [6,27,28]. The conformational change required for AMPK activation can also be induced by compounds that act as AMP analogs and therefore under conditions that do not involve changes in the ratio of AMP/ATP. AICAR, a nucleoside widely used as AMPK activator, is converted inside the cell to its mono-phosphorylated form ZMP (5-amino-4-imidazolecarboxamide ribotide), and as such behaves as an AMP analogue capable of activating AMPK upstream of LKB1 [9]. AICAR mediated AMPK activation has been reported to inhibit cell proliferation and cell cycle progression via inhibition of the PI3K/Akt pathway and the cell cycle regulatory proteins p21, p27 and p53 [16].

In the present study we have investigated the effect of AMPK activation by AICAR on the proliferation, cell cycle progression and apoptosis of various childhood ALL cell models characterized by non-random translocation signature profiles, and representing chemotherapy sensitive and resistant phenotypes [29-32]. AICAR-mediated AMPK activation was found to be a proficient cytotoxic agent in ALL cells and the mechanism of its anti-proliferative and apoptotic effect appear mediated via activation of p38-MAPK pathway, increased expression of cell cycle inhibitory proteins p27 and p53, and downstream effects on the mTOR pathway, hence exhibiting therapeutic potential as a molecular target for the treatment of childhood ALL.

## Materials and methods

### Materials

RPMI 1640 medium was obtained from Mediatech, Inc. (Herndon, VA). Iscove's modified Dulbecco medium (IMDM) and fetal bovine serum (FBS) were obtained from GIBCO/Invitrogen (Carlsbad, CA). AICAR was purchased from Toronto Research Chemicals (Ontario, Canada). Iodotubericidin and SB 202190 were obtained from Calbiochem (San Diego, CA). CellTiter 96 Aqueous One Solution Cell Proliferation Assay kit was purchased from Promega (Madison, WI). [^3^H]Thymidine ribotide ([^3^H]TdR) was purchased from Amersham Biosciences (GE Healthcare, Piscataway, NJ). The Propidium Iodide (PI)-RNase Staining kit was obtained from BD Pharmigen (Franklin, NJ). The enhanced chemiluminescence (ECL) detecting reagent was from Amersham Biosciences. Primary antibodies against p21, p27, and p53 were purchased from Santa Cruz Biotechnology, Inc. (Santa Cruz, CA). Antibodies against phosphospecific as well as pan-Akt, AMPK and p38-MAPK were obtained from Cell Signaling (Beverly, MA).

### Childhood ALL Cell Lines

The following childhood ALL cell lines were used in this study: CCRF-CEM (T-lineage ALL), NALM6 (B-lineage precursor Bp-ALL), REH (Bp-ALL expressing the TEL/AML1 fusion protein) as representative of a chemotherapy sensitive phenotype, and SupB15 (Bp-ALL expressing the BCR/ABL fusion protein) as a model of a chemotherapy resistant phenotype. CCRF-CEM, REH and SupB15 cells were obtained from ATCC (Rockville, MD), NALM6 cells were purchased from DSMZ (Braunschweig, Germany).

### Tissue Culture

The childhood ALL cell lines CCRF-CEM, NALM6 and REH were maintained in RPMI 1640 medium supplemented with 10% FBS and antibiotics as described elsewhere [33]. SupB15 cells were maintained in IMDM medium with 20% FBS. All cells were grown at 37°C and 5% CO_2 _atmosphere, and all drug treatments were done in the presence of serum.

### Cell Proliferation Assay

Cell growth and viability were assessed using the tetrazolium compound [3-(4,5-dimethylthiazol-2-yl)-5-(3-carboxymethoxyphenyl)-2-(4-sulfophenyl)-2H-tetrazolium inner salt] (MTS) (Promega). Briefly, 0.5 × 10^6 ^cells/well of each cell lines were plated and incubated for 18 – 24 h with or without AICAR at various concentrations. Next, 20 μl of MTS solution was added to each well and cells were incubated for an additional 2 to 4 h, after which, absorbance at 490 nm was determined using a microplate reader as a reflection of MTS reduction by viable cells. Values were expressed as a percentage relative to those obtained in untreated controls.

### Thymidine Incorporation Assay

Proliferation of cells was also determined by [^3^H]thymidine ribotide ([^3^H]TdR) incorporation into DNA. Each cell line was plated at a density of 0.25 × 10^6 ^cells/well, and cells were incubated for 18–24 h with or without AICAR at various concentrations and then exposed to 37 kBq/ml [*methyl*-^3^H]thymidine for 6 h. Suspension cell cultures were harvested using a cell harvester (Packard instrument Co., Meriden, CT), and radioactivity was measured using a 1450 microbeta liquid scintillation counter (PerkinElmer Life Sciences).

### Flow Cytometry Assessment of Cell Cycle

Cells were cultured in 6-well plates and treated with AICAR prior to assessment of cellular DNA content by flow cytometry. Following treatment, cells were washed with PBS, and 1.0 × 10^6 ^cells were resuspended in 100 μl of PBS, and 5 ml of 70% ethanol were added slowly, while under continuous vortexing, for fixation overnight. Subsequently, cells were washed, and suspended in 500 μl of PI/RNase solution and cell cycle progression was determined by flow cytometry (BD Biosciences FACSCalibur flow cytometer) using the Modfit LT software.

### Apoptosis/DNA Ladder Gel Assay

Ten million purified ALL cells were obtained by centrifugation after exposure to 0.1 to 1.0 mM AICAR for 48 h. Cells were lysed in 0.5 ml of 20 mM Tris (pH 7.4), 0.4 mM EDTA, 0.25% Triton X-100 (American Bioanalytical, Natick, MA). After 15 min of incubation at room temperature, nuclei were removed by centrifugation at 14,000 rpm (RCF = 16,000). The supernatant was transferred to a new tube and nuclear DNA was precipitated overnight at -20°C using 55 μl of 5 M NaCl and 550 μl of isopropanol. After centrifugation at 14,000 rpm for 10 minutes, the pellet was washed with 70% ethanol and resuspended in 20 μl of 10 mM Tris (pH 8.0), 1 mM EDTA, and 0.1 mg/ml RNase. The DNA preparations were separated by 1.6% Tris borate EDTA agarose gel electrophoresis and visualized by ethidium bromide staining.

### Immunoblot

After stipulated incubation times in the presence or absence of AICAR, cells were harvested, washed with PBS, and sonicated in 50 mM Tris-HCl (pH 7.4) containing protease inhibitors (1 mM phenylmethylsulfonyl fluoride, 5 μg/ml aprotinin, 5 μg/ml antipain, 5 μg/ml pepstatin A, and 5 μg/ml leupeptin). Proteins (50 μg/lane) were resolved by SDS-PAGE and transferred onto nitrocellulose membranes. The membranes were blocked for 1 h in 5% nonfat dry milk in TTBS (20 mM Tris, 500 mM NaCl, and 0.1% Tween 20, pH 7.5) and incubated overnight in primary antibody (p21, p27, p53, AMPK, p38-MAPK, Akt, β-actin, at a 1:2000 dilution) containing 5% nonfat dry milk for non-phospho antibodies and containing 5% albumin for phospho-antibodies (P-Akt, P-AMPK, P-p38-MAPK, at a 1:1000 dilution). The blots were washed four times (5 min) with TTBS and incubated for 45 min at room temperature with the respective horseradish peroxidase-conjugated secondary antibody (1:5000). The blots were washed three times in TTBS and once in 0.1 M PBS (pH 7.4) at room temperature, and protein expression levels determined using the ECL detection kit (Amersham Biosciences). The relative integrated density value (IDV) of each immunodetected band was determined using the ChemiDoc XRS digital imaging system with the Quantity One 1-D Analysis Software Version 4.6.3 (Bio-Rad Laboratories, Inc., Hercules, CA). The IDV data were normalized to β-actin levels and expressed relative to control.

### Statistical Analysis

Multiple comparisons of cell proliferation, and number of viable cells (%) were assessed by one-way ANOVA followed by the Newman-Keuls multiple comparison test. Individual comparisons were achieved using one-tailed, unpaired *t *test according to the Graph Pad PRISM software version 2 (GraphPad Software, Inc., San Diego, CA). The data were expressed as mean ± SEM.

## Results

### AICAR induces growth inhibition in ALL cells via AMP-activated protein kinase (AMPK)

**Figure 1:**
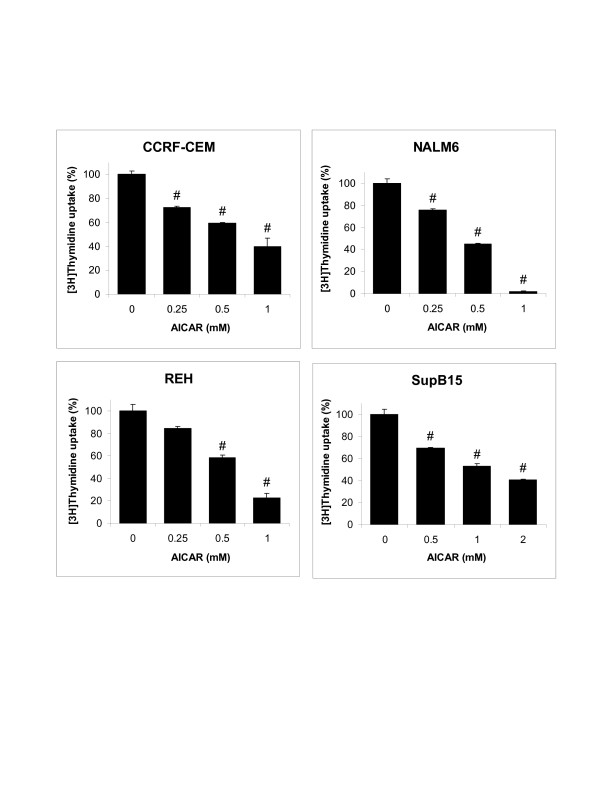
AICAR treatment inhibits proliferation of human childhood leukemia ALL cells. The ALL cell lines CCRF-CEM (T-lineage), NALM6 (Bp-lineage), REH (Bp-ALL expressing the TEL/AML1 fusion protein), and SupB15 (Bp-ALL expressing the BCR/ABL fusion protein) were treated for 24 h with various concentrations of AICAR (0.25 – 2 mM), and cell growth analyzed by thymidine ribotide ([^3^H]TdR) incorporation into DNA. The results are expressed as percentage of [^3^H]thymidine uptake (%) relative to control values (mean ± SEM, n = 3). #, *p *< 0.001 for AICAR-treated cells *vs. *control.

We investigated the effect of AICAR on the growth of CCRF-CEM, NALM6, REH, and SupB15 ALL cells. REH and SupB15 cell lines were used as models for B-precursor ALL sensitive and resistant phenotypes, respectively. Cells were treated with various concentrations of AICAR (0.25 to 1.0 mM) for 24 h, and growth was examined by [^3^H]thymidine uptake assays. As shown in Figure 1, AICAR inhibited the growth of CCRF-CEM, NALM6, and REH in a dose-dependent manner at all doses studied (*p *< 0.001, cells treated with AICAR *vs. *untreated/control cells). SupB15 cells, which harbor the BCR/ABL translocation, were relatively resistant to lower concentrations of AICAR, but exhibited significant growth inhibition when treated with higher concentrations of AICAR (*p *< 0.001, cells treated with 0.5 to 2.0 mM AICAR *vs. *untreated/control cells).

**Figure 2:**
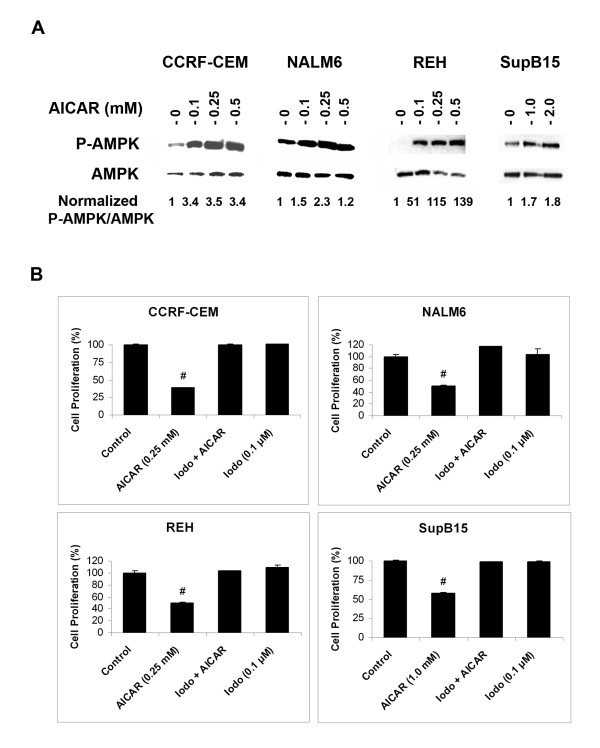
The anti-proliferative effect of AICAR on ALL cells is mediated via activation of AMPK. (**A**) Western blot analysis of phosphorylated AMPK (P-AMPK, Thr172) expression in CCRF-CEM, NALM6, REH, and SupB15 cells treated with various concentrations of AICAR (0 – 2.0 mM). Total protein was extracted from AICAR-treated cells and AMPK and P-AMPK were immunodetected using specific antibodies. Equal amounts of protein (50 μg) were loaded per lane as confirmed by β-actin level. Density value of P-AMPK bands were normalized to level of AMPK and expressed relative to control. (**B**) Cell proliferation assays of ALL cells treated for 18 h with AICAR alone (0.25 mM for CCRF-CEM, NALM6, REH, and 1.0 mM for SupB15), the adenosine kinase inhibitor iodotubericidin alone (Iodo, 0.1 μM), or both agents together (Iodo + AICAR). Growth inhibition was determined using the tetrazolium (MTS) reduction assay. Values are expressed as a percentage relative to those obtained with untreated control cells (mean ± SEM). Data are representative of at least three independent experiments. #, *p *< 0.001 for AICAR *vs. *Iodo + AICAR.

To investigate whether the anti-proliferative effect of AICAR on ALL cells is mediated via AMPK activation, the phosphorylation of AMPK was examined. AICAR induced the phosphorylation of AMPK in a dose-dependent manner in CCRF-CEM, NALM6, and REH cell lines (Figure 2A). The highest effect on AMPK activation was observed with REH cells, which express the TEL/AML1 fusion protein (139 fold in cells treated with 0.5 mM AICAR as compared to untreated/control cells). Again, in SupB15 (BCR/ABL) cells, AMPK activation by AICAR was seen at higher concentrations (1.0 and 2.0 mM) than those required to induce AMPK phosphorylation in the other cell lines tested. To confirm that the anti-proliferative effects observed in ALL cells resulted from AICAR induced activation of AMPK, we used the adenosine kinase inhibitor iodotubericidin (Iodo). This compound blocks AMPK activation by preventing the intracellular conversion of AICAR to its active metabolic form, ZMP [34]. The CCRF-CEM, NALM6, REH, and SupB15 cells were pretreated with iodotubericidin (0.1 μM) for 30 min prior to the addition of AICAR (0.25 mM with the exception of 1 mM for SupB15), and cell proliferation was measured after 18 h with the tetrazolium (MTS) reduction assays. As shown in Figure 2B, pre-treatment with iodotubericidin resulted in abrogation of the anti-proliferative activity of AICAR in all ALL cell lines studied, while no effect was seen in untreated controls or cells treated with iodotubericidin alone (*p *< 0.001, cells treated with AICAR *vs. *IODO + AICAR). Taken together, these results indicate that the anti-proliferative activity of AICAR in ALL cells is mediated by P-AMPK.

### AICAR-induced regulation of cyclin dependent kinase inhibitors leads to cell cycle arrest and AMPK activation dependent apoptosis in ALL cells

**Figure 3:**
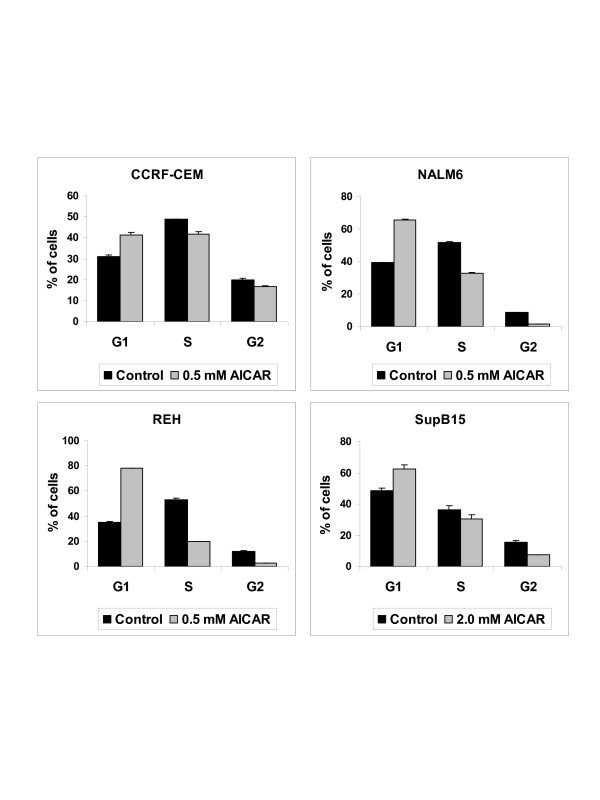
AICAR treatment induces cell cycle arrest in G1-phase in ALL cells. DNA content of CCRF-CEM, NALM6, REH, and SupB15 ALL cells untreated (Control) or treated with AICAR (0.5 and 2.0 mM) for 48 h was measured by fluorescence-activated cell sorting (FACS) using propidium iodide staining. The panels represent distribution of cells (%) in G1-, S-, and G2-phase of the cell cycle obtained from FACS analysis. Data are representative of at least three independent experiments and values are expressed as mean ± SEM.

**Figure 4:**
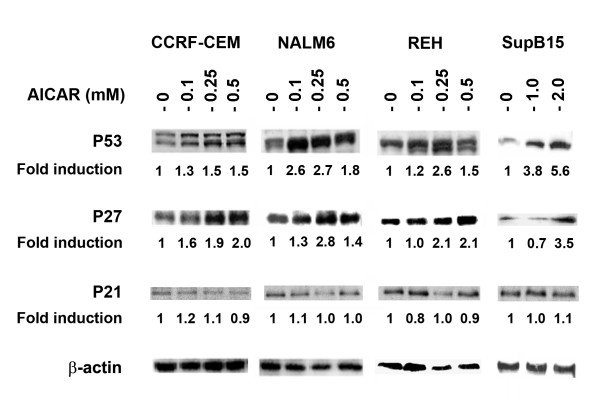
AICAR induces upregulation of the p53 and the cyclin dependent kinase inhibitor p27 in ALL cells. Western blot analyses of p53, p27, and p21 were done using cell extracts from CCRF-CEM, NALM6, REH, and SupB15 cells treated with the indicated concentrations of AICAR (0 – 2 mM) for 48 h. Equivalent amount of proteins (50 μg) were separated by SDS-PAGE and immunodetected with antibodies against p53, p27, and p21. Membranes were stripped and reprobed with anti-β-actin antibody to confirm equal amount of proteins loaded in all lanes. Density value of each band was normalized to their respective β-actin level and expressed relative to control (untreated). The data shown are representative of 3 experiments producing similar results.

To examine cell cycle progression of ALL cells during treatment with AICAR, we exposed cells to AICAR for 48 h prior to flow cytometry analysis. Treatment with AICAR at 0.5 mM resulted in increased of CCRF-CEM, NALM6, and REH cells arrested in G1-phase (Figure 3). For SupB15 cells, higher concentrations of AICAR (> 1 mM) were required to induce similar arrest in G1-phase. To further investigate the effect of AICAR induced cell cycle arrest in G1-phase, we examined the expression of specific cell-cycle inhibitors such as the cyclin-dependent kinase (cdk) inhibitors p21 and p27, which bind to cyclin-cdk complexes and inhibit the progression of cell cycle [35]. We also examined the expression of p53, one of the major cellular checkpoint proteins [36]. As shown in Figure 4, AICAR induced the expression of the cip/kip protein cdk inhibitor p27 in CCRF-CEM, NALM6, REH, and SupB15 cells in a dose-dependent manner, but had little or no effect on the expression of the cell-cycle protein inhibitor p21 (Figure 4). In addition, AICAR induced the expression of the tumor suppressor gene p53 suggesting a role for p53 in the AMPK signaling pathway. As expected, no change was observed in the level of β-actin gene expression in both untreated- and treated-cells.

**Figure 5:**
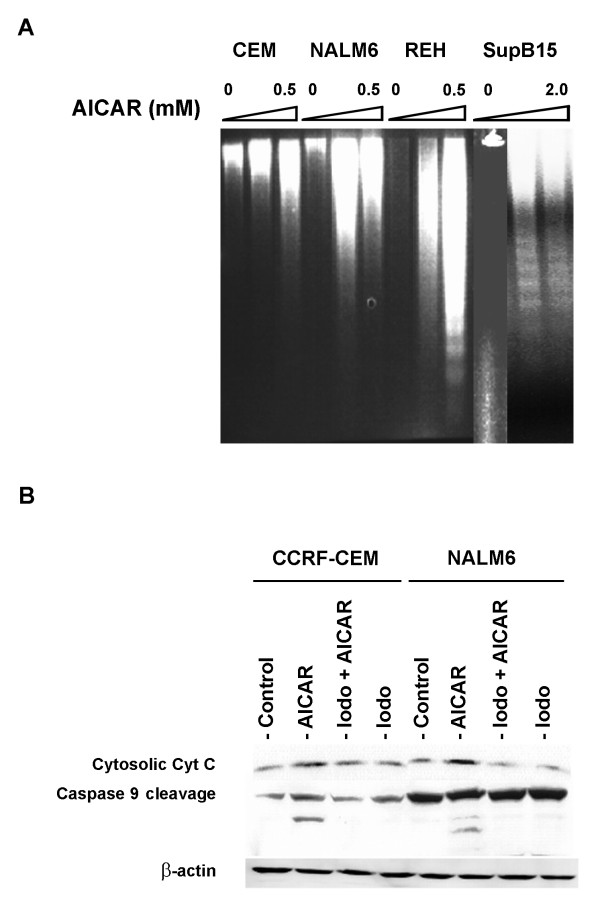
AICAR-activation of AMPK mediates apoptosis in ALL cells via the mitochondrial pathway. (**A**) DNA fragmentation assay. CCRF-CEM, NALM6, REH, and SupB15 cells were treated for 48 h with increased concentrations of AICAR (0 – 2 mM), and nuclear DNA was analyzed by electrophoresis on a 1.6% Tris-Borate-EDTA agarose gel. (**B**) Western blot analysis of cytochrome C and caspase 9 expression in CCRF-CEM and NALM6 cells treated for 48 h with 0.5 mM AICAR alone, 0.1 μM iodotubericidin alone (Iodo), or both agents together (Iodo + AICAR). Equal amount of loaded protein (50 μg) was confirmed by immunoblotting with anti-β-actin antibody. The data shown are representative of 3 experiments.

We then tested if the growth inhibition and cell cycle arrest induced by AICAR in ALL cell lines led to apoptosis. For this, the ALL cells were treated with 0.1 up to 2.0 mM AICAR for 48 h and nuclear DNA fragmentation was assessed. As shown in Figure 5A, treatment with 0.5 mM AICAR consistently induced DNA fragmentation in CCRF-CEM, NALM6, and REH cells, while for SupB15 cells higher concentrations were required (>1 mM). We also evaluated the expression of two apoptotic regulatory proteins, cytochrome C and caspase 9 [37], in CCRF-CEM and NALM6 cells following treatment with 0.5 mM AICAR. In both cell lines, AICAR resulted in increased levels of cytochrome C release and caspase 9 cleavage (Figure 5B). This effect was blocked by addition of the adenosine kinase inhibitor iodotubericidin (Figure 5B, Iodo + AICAR). On the other hand, cells treated with 0.1 μM iodotubericidin alone (Iodo) exhibited levels of cytochrome C and caspase 9 comparable to untreated cells (Control). These phenotypes of apoptosis observed in CCRF-CEM and NALM6 upon treatments with AICAR correlated with activation of P-AMPK (Figure 2A). Therefore, these results suggest that apoptosis induced by AICAR-activation of AMPK in ALL cell lines is mediated via the mitochondrial pathway.

### Anti-proliferative action of AICAR on ALL cells is associated with downstream AMPK-dependent activation of p38-MAPK

**Figure 6:**
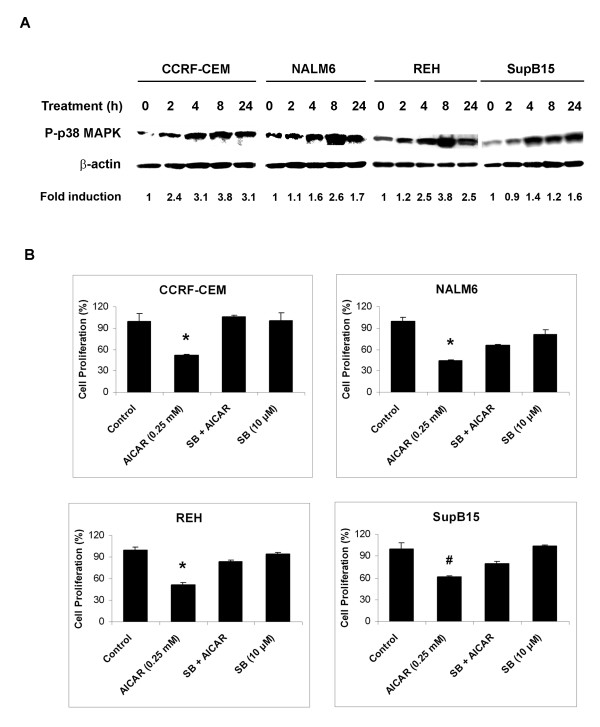
Anti-proliferative action of AICAR on ALL cells is associated with downstream AMPK-dependent activation of p38-MAPK. (**A**) CCRF-CEM, NALM6, REH, and SupB15 ALL cells treated with 0.25 mM AICAR for the indicated times (0 – 24 h) were analyzed by Western blot for phosphorylated p38-MAPK protein (P-p38-MAPK, Thr180/Tyr182). β-actin was used as a loading control. Density value of each band was normalized to their respective β-actin level and expressed relative to control (untreated). (**B**) Cell proliferation assays of CCRF-CEM, NALM6, REH, and SupB15 cells treated with 0.25 mM AICAR alone, 10 μM of the p38-MAPK inhibitor SB 202190 alone (SB), or both agents together (SB + AICAR). The cell proliferation values are expressed as a percentage relative to those obtained with untreated control cells (mean ± SEM, n = 3). Data are representative of at least three independent experiments. *, *p *< 0.01 for AICAR *vs. *SB + AICAR; #, *p *< 0.05 for AICAR *vs. *SB + AICAR.

Involvement of mitogen-activated protein kinases (MAPKs) is one of the most relevant aspects in the regulation of cell cycle and apoptosis [38,39]. In order to investigate if MAPKs were involved in the anti-proliferative activity of AICAR, we determined the phosphorylation levels of p38-MAPK in ALL cells following treatment with AICAR. This was done by Western blot analysis of protein extracts obtained from CCRF-CEM, NALM6, REH, and SupB15 cells treated with 0.25 mM AICAR for 2, 4, 8, and 24 h. Figure 6A shows that AICAR induced phosphorylation of p38-MAPK in a time dependent manner. The involvement of p38-MAPK in AICAR mediated cytotoxicity was further examined using the p38-MAPK inhibitor SB 202190 [40,41]. ALL cells were pretreated with 10 μM SB 202190 for 30 min before the addition of 0.25 mM AICAR, and cell proliferation was measured after 18 h using cell proliferation assays. Pretreatment of ALL cells by SB 202190 prevented the proliferation arrest caused by AICAR in all cell lines studied (*p *< 0.05, AICAR *vs. *SB + AICAR) (Figure 6B, SB + AICAR) whereas treatments with 10 μM SB 202190 alone (SB) or 0.1% DMSO (Control) had no effect on cell growth. Therefore, our results strongly suggest that phosphorylation of p38-MAPK (P-p38-MAPK) is an important step towards inhibition of cell proliferation associated with AICAR treatment in ALL cells.

**Figure 7:**
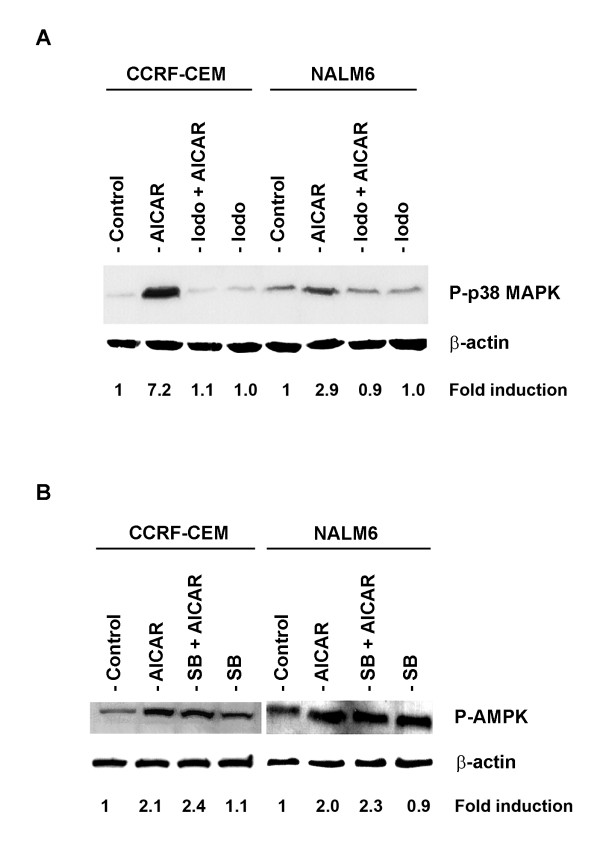
Activation of AMPK is required for phosphorylation of p38-MAPK in AICAR-treated ALL cells. (**A**) Immunoblot of p38-MAPK phosphorylation (P-p38-MAPK, Thr180/Tyr182) and β-actin (loading control) expressed in CCRF-CEM and NALM6 cells treated with 0.1% DMSO (Control), 0.5 mM AICAR alone, 0.1 mM iodotubericidin alone (Iodo), or both agents together (Iodo + AICAR). (**B**) Phosphorylation status of AMPK (P-AMPK, Thr172) from CCRF-CEM and NALM6 cells incubated with either 0.5 mM AICAR alone, 10 μM SB 202190 alone (SB) or both inhibitors together (SB + AICAR). Level of β-actin was used as loading controls. Density value of each band was normalized to their respective β-actin level and expressed relative to control. The immunoblots shown are representative of 3 independent experiments, which produced similar results.

In order to investigate the sequence of events of AMPK and p38-MAPK activation in ALL cells following treatment with AICAR, we first determined whether phosphorylation of p38-MAPK required activation of AMPK by AICAR using the specific AICAR inhibitor iodotubericidin, and thereafter evaluated the effect of the p38-MAPK inhibitor SB 202190 on AMPK activation. We reasoned that if activation of AMPK was required for induction of P-p38-MAPK, blocking its activation should result in non-phosphorylation of p38-MAPK and therefore reversal of the anti-proliferative effect. Indeed, pre-treatment of CCRF-CEM and NALM6 cells with 0.1 mM iodotubericidin (Iodo) prior to AICAR treatment (0.25 mM) blocked phosphorylation of p38-MAPK (Figure 7A, Iodo + AICAR), whereas treatment with 0.1% DMSO (Control) or iodotubericidin alone had no influence on the level of P-p38-MAPK. As expected, treatment with AICAR increased the level of P-p38-MAPK by 7.2- and 2.9-fold in CCRF-CEM and NALM6 cells, respectively, when compared to control (Figure 7A). Conversely, pre-incubation of CCRF-CEM and NALM6 cells with SB 202190 (10 μM) prior to AICAR treatment did not inhibit AMPK activation (Figure 7B, SB + AICAR). Expression of P-AMPK level was similar between AICAR- and SB + AICAR-treated cells (2.1- and 2.4-fold in CCRF-CEM, and 2.0- and 2.3-fold in NALM6 cells). These data indicate that activation of AMPK is required for phosphorylation of p38-MAPK in AICAR-treated ALL cells.

### AICAR treatment in ALL cells results in activation of Akt

**Figure 8:**
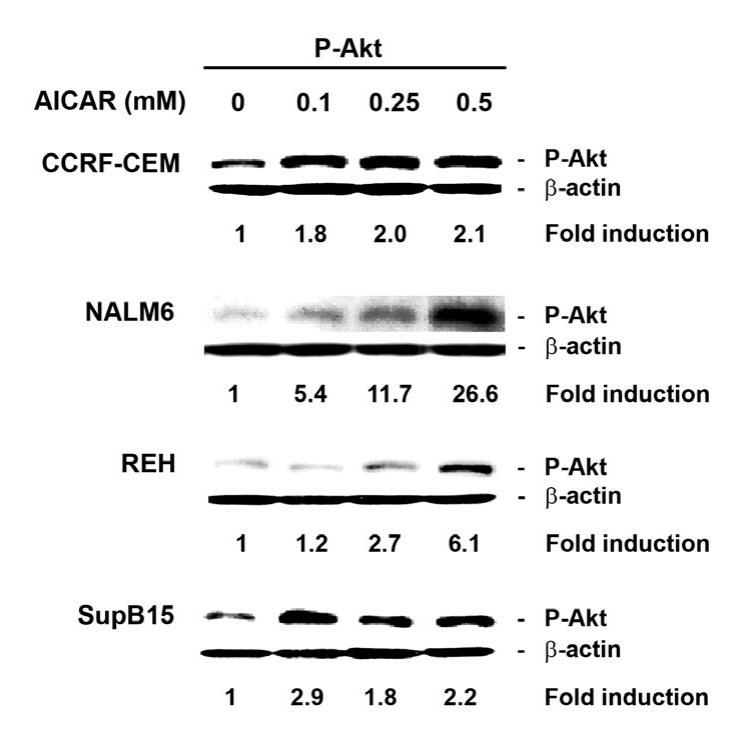
AICAR treatment on ALL cells results in activation of Akt. Western blot analysis depicting the level of phosphorylated Akt protein (P-Akt, Ser473) in CCRF-CEM, NALM6, REH, and SupB15 cells were treated for 24 h with various concentrations of AICAR (0 – 0.5 mM). β-actin was used as control for the amount of proteins loaded per lane. Density value of each band was normalized to their respective β-actin level and expressed relative to control (untreated). The immunoblots shown are representative of at least 3 independent experiments.

It has been shown that AMPK also regulates the mTOR pathway through activation of TSC2 [24]. Since TSC2 is also regulated by Akt, a key downstream regulator of the PI3K pathway that is important for cell growth, proliferation, and survival [42,43], we investigated the role of PI3K/Akt signaling on AICAR-mediated AMPK activation. For this, we evaluated the level of phosphorylated Akt (P-Akt) protein in CCRF-CEM, NALM6, REH, and SupB15 ALL cells treated with 0.5 mM AICAR using Western blot. As shown in Figure 8, cells treated with increasing concentrations of AICAR exhibited higher levels of P-Akt as compared to untreated/control cells, and this effect was dose-dependent. This result suggests that activation of the cell survival PI3K/Akt signaling pathway may be used as compensatory survival mechanism against AICAR mediated cytotoxicity.

### The anti-proliferative activity of AICAR is enhanced by the addition of the mTOR inhibitor rapamycin in ALL cells

**Figure 9:**
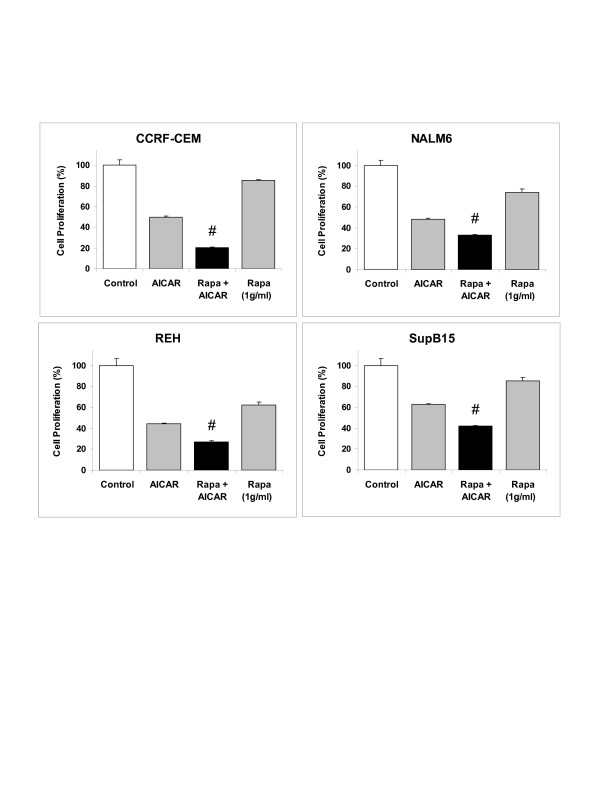
The anti-proliferative activity of AICAR is enhanced by the addition of the mTOR inhibitor rapamycin in ALL cells. CCRF-CEM, NALM6, REH, and SupB15 ALL cells were incubated for 24 h with either AICAR alone (0.25 mM for CCRF-CEM, NALM6, REH, and 1.0 mM for SupB15), 1 μg/ml rapamycin alone, or a combination of both drugs (Rapa + AICAR) and cell proliferation determined using the tetrazolium (MTS) reduction assays. The cell proliferation values are expressed as a percentage relative to those obtained with untreated control cells (mean ± SEM, n = 3). #, *p *< 0.005 for AICAR *vs. *Rapa + AICAR.

Based on the observation that Akt is activated following AICAR treatment in ALL cells, we hypothesized that inhibition of the PI3K/Akt pathway through inactivation of mTOR using rapamycin should enhance the cytotoxic activity of AICAR. To test this hypothesis, we treated the ALL cells lines CCRF-CEM, NALM6, and REH with 0.25 mM AICAR alone, 1 μg/ml rapamycin alone (Rapa), or a combination of both agents (rapamycin and AICAR) for 24 h. For SupB15 cells, a higher concentration of AICAR was used (1 mM). As seen in Figure 9, treatment of the cells with AICAR as single agent resulted in 40–50% growth inhibition, while rapamycin alone inhibited 20–30% of growth as compared to untreated cells. Combination of the two agents significantly increased the level of growth inhibition by 60–80% in all four ALL cell lines tested (*p *< 0.005). These results indicate that targeted therapy using combination of drugs that target both the AMPK signaling and the PI3K/Akt/mTOR pathway may be a useful strategy in childhood ALL.

## Discussion

**Figure 10:**
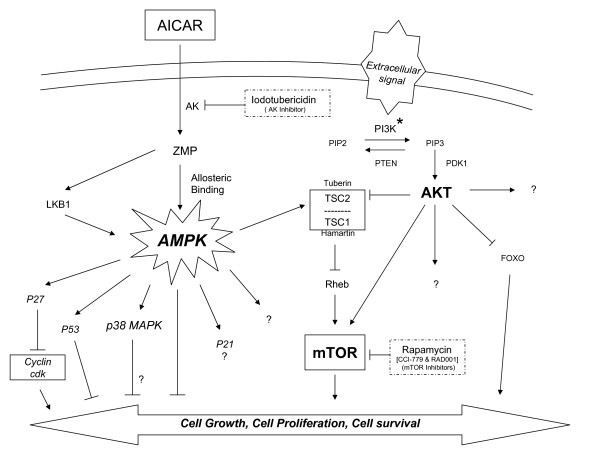
Proposed mechanism of action for AICAR in human leukemia ALL cells. After crossing the cell membrane, AICAR is metabolized by the adenosine kinase (AK) to its active mono-phosphorylated form ZMP (AMP analogue) which activates AMPK. The activated AMPK will signal to multiple downstream targets impinging on cell growth, cell proliferation, and cell survival. In ALL cells, induction of AMPK by AICAR increases the levels of p53, the cdk inhibitor p27, and the p38-MAPK leading to inhibition of cell proliferation, cell arrest in G1-phase, and apoptosis. In addition, P-AMPK activates TSC2 reducing the level of mTOR, an important regulatory factor of the PI3K/Akt pathway necessary for cell proliferation [24]. As a cell survival mechanism, the level of Akt is increased to overcome the reduction in mTOR attempting to restore or promote cell growth. AICAR, 5-Aminoimidazole-4-Carboxamide-1-β-4-Ribofuranoside; AK, adenosine kinase; AMPK, AMP activated protein kinase; PI3K, phosphatidylinositol 3-kinase; Akt, proteinase kinase B; TSC, tuberous sclerosis complex; mTOR, mammalian target of rapamycin; MAPK, mitogen-activated protein kinase.

In the present study we demonstrated that AMPK activation by AICAR induces growth inhibition, G1-phase cell cycle arrest and apoptosis in childhood ALL cell lines. Our data indicate that growth inhibition and apoptosis are directly mediated by AMPK activation and that induction of G1 cell cycle arrest in ALL cells involves phosphorylation of p38-MAPK and upregulation of the cdk inhibitor p27 and p53 (see proposed model Figure 10). Furthermore, treatment of ALL cell lines with AICAR led to the phosphorylation of Akt and activation of the PI3K/Akt/mTOR pathway, and that combination of AICAR and the mTOR inhibitor rapamycin resulted in increased cytotoxicity compared to treatment with each agent alone.

Multiple studies have demonstrated that a key function of AMPK is regulation of the intracellular energy balance. This is supported by AMPK's activation in response to low levels of ATP [1,2]. AMPK activation in turn results in phosphorylation of multiple downstream targets to switch off ATP-consuming (fatty acid and cholesterol synthetic pathways) pathways and to turn on ATP-generating pathways (glycolysis and fatty acid oxidation) [5]. The end result is protein synthesis, cell growth, proliferation and survival. Several observations support this role of AMPK in cancer cells [44,45]. Indeed, many cancers have increased expression of enzymes that are inhibited by AMPK, such as FAS and mTOR [17,46]. Paradoxically, when activated pharmacologically, AMPK is able to induce apoptosis in various tumor cell types [10,16]. Two tumor suppressors gene products have been identified as the upstream activator and downstream effector of AMPK, namely LKB1 and TSC2, respectively [6,18,19]. A small, but emerging, body of literature demonstrates that AMPK activation is capable of inhibiting growth in cancer cell *in vitro *[16,17,47]. The mechanisms responsible for these opposing effects of AMPK activation are yet to be fully understood but the anti-proliferative and pro-apoptotic effect of AMPK has been shown to be mediated via the negative regulation of mTOR by LKB1 [48]. Others have also reported that AMPK's growth inhibitory properties are mediated by various other mechanisms, including inhibition of *de novo *fatty acid synthesis and p70S6K mediated inhibition of protein synthesis, inhibition of cell cycle progression by p21, and attenuation of PI3K and Akt pathways [17].

Although advances in the treatment of children with ALL have resulted in survival rates approaching 90% for all subtypes combined, outcome for patients diagnosed with resistant phenotypes and for those who relapse continues to be dismal [29-32]. Consequently, ALL continues to be a leading cause of cancer related death in children and adolescents, underscoring the need to discover new targets and develop novel treatment strategies for patients with this disease [14]. Our results suggest that AMPK is one such target, and that its activation by the nucleoside AICAR is an efficient strategy to induce apoptosis in childhood ALL cells. AICAR induced growth inhibition as determined by [^3^H]thymidine incorporation assays in a dose dependent manner in all ALL cell lines studied. Further, AICAR was also capable of inducing growth inhibition in those ALL cell lines representative of more resistant phenotypes, such as CCRF-CEM (T-ALL) and SupB15 (BCR/ABL positive Bp-ALL), even though higher concentrations of AICAR were required to inhibit the latter cell type. Although AMPK-independent effects have been reported for AICAR [17], our data demonstrate that its growth inhibitory effects were mediated via phosphorylation of AMPK as confirmed by the ability of the adenosine kinase inhibitor iodotubericidin to reverse the cytotoxic effects of AICAR.

AMPK activation by AICAR has also been reported to induce cell cycle arrest in cancer cell culture models [16]. It has been shown that AMPK activation induces the expression of wild-type p53 and p21 in rat hepatoma cells [16]. Most reports indicate AMPK activation induces apoptosis by increasing the phosphorylation of p53 at Ser15 [49-51]. The upstream activator of AMPK has also been implicated in increasing the concentration of p21 when transfected in LKB1-deficient A549 lung adenocarcinoma cells [52]. Others have reported cell cycle arrest in S-phase after AMPK activation by AICAR using C6 glioma and U87MG astrocytic cell lines [16]. Our data are consistent with these reports, as AICAR induced cell cycle arrest in G1-phase in all lymphoid leukemia cell lines we examined, including the BCR/ABL positive cell line SupB15. Although we also observed an increase in p53 expression leading to cell cycle arrest in these cell lines, no consistent change in the level of p21 expression was detected. In contrast, our data demonstrate increased expression of another cell cycle regulatory protein, p27. The TSC proteins tuberin and hamartin are known to be positive regulators of the cyclin dependent kinase p27 and tuberin (TSC2) has been reported to protect the ubiquitin-dependent degradation of p27 [53,54]. In addition, it has been shown that tuberin can affect p27 localization as p27 is translocated to the nucleus [55,56]. Our data also demonstrate increase phosphorylation of the mitogen-activated protein kinase p38-MAPK. Several reports indicate that activation of p38-MAPK results in inhibition of the cell cycle at the G1/S boundary, such as it was seen in ALL cells after AICAR mediated activation of AMPK (and increased P-p38-MAPK). The exact mechanism leading to p53 and p27 mediated cell cycle arrest in ALL cell line following activation of AMPK is not fully understood and it is currently under investigation in our laboratory.

AICAR induced phosphorylation of AMPK triggered apoptosis in all cell lines studied, although higher concentrations of AICAR were required for SupB15 cells. Both cytochrome C release and caspase 9 cleavage were observed in NALM6 and CCRF-CEM cells treated with AICAR, and co-incubation with the inhibitor of AICAR metabolism to its activated form ZMP, iodotubericidin, was able to block these apoptotic effects. While others have reported concomitant inhibition of the PI3K/Akt pathway after exposure to AICAR [16], we consistently observed increase phosphorylation of Akt in ALL cell lines treated with AICAR. It is known that AMPK and Akt have opposite regulatory effects on the mTOR pathway [57]. Activated AMPK via its downstream effector TSC2 negatively regulates mTOR, leading to apoptosis, while Akt promotes activation of the mTOR pathway [24,43]. We interpreted the consistent activation of Akt in ALL cell lines after AICAR induced AMPK activation as a potentially compensatory pro-survival mechanism. Therefore, we hypothesized that targeting the mTOR pathway while simultaneously activating AMPK should result in increased cytotoxicity. Indeed, our data demonstrate that the combination of AICAR and rapamycin resulted in additive growth inhibitory effects in all ALL cell lines studied. These results suggest simultaneous activation of AMPK and inhibition of the PI3K/Akt/mTOR pathway is an attractive combination targeted therapy for the treatment of childhood ALL leukemia, and may be active in the treatment of resistant phenotypes.

## Authors' contributions

TKS carried out cell cultures, cell proliferation and apoptosis assays, flow cytometry analysis, Western blots, conceived the study, participated in its design, and drafted the manuscript. GML determined the relative density values of each immunodetected band, analyzed the data, performed the statistical analysis, and drafted the manuscript. TTHK performed Western blots. GJL participated in the design of the study, and contributed to the final drafting and critical revision of the manuscript. IS conceived the study. JCB conceived the study, participated in its design and coordination, and contributed to the final drafting and critical revision of the manuscript. All authors read and approved the final manuscript.

## References

[B1] Hardie DG, Carling D (1997). The AMP-activated protein kinase--fuel gauge of the mammalian cell?. Eur J Biochem.

[B2] Kemp BE, Mitchelhill KI, Stapleton D, Michell BJ, Chen ZP, Witters LA (1999). Dealing with energy demand: the AMP-activated protein kinase. Trends Biochem Sci.

[B3] Hardie DG, Scott JW, Pan DA, Hudson ER (2003). Management of cellular energy by the AMP-activated protein kinase system. FEBS Lett.

[B4] Davies SP, Carling D, Hardie DG (1989). Tissue distribution of the AMP-activated protein kinase, and lack of activation by cyclic-AMP-dependent protein kinase, studied using a specific and sensitive peptide assay. Eur J Biochem.

[B5] Kemp BE, Stapleton D, Campbell DJ, Chen ZP, Murthy S, Walter M, Gupta A, Adams JJ, Katsis F, van Denderen B, Jennings IG, Iseli T, Michell BJ, Witters LA (2003). AMP-activated protein kinase, super metabolic regulator. Biochem Soc Trans.

[B6] Hawley SA, Boudeau J, Reid JL, Mustard KJ, Udd L, Makela TP, Alessi DR, Hardie DG (2003). Complexes between the LKB1 tumor suppressor, STRAD alpha/beta and MO25 alpha/beta are upstream kinases in the AMP-activated protein kinase cascade. J Biol.

[B7] Fryer LG, Parbu-Patel A, Carling D (2002). The Anti-diabetic drugs rosiglitazone and metformin stimulate AMP-activated protein kinase through distinct signaling pathways. J Biol Chem.

[B8] Sullivan JE, Brocklehurst KJ, Marley AE, Carey F, Carling D, Beri RK (1994). Inhibition of lipolysis and lipogenesis in isolated rat adipocytes with AICAR, a cell-permeable activator of AMP-activated protein kinase. FEBS Lett.

[B9] Corton JM, Gillespie JG, Hawley SA, Hardie DG (1995). 5-aminoimidazole-4-carboxamide ribonucleoside. A specific method for activating AMP-activated protein kinase in intact cells?. Eur J Biochem.

[B10] Kefas BA, Cai Y, Ling Z, Heimberg H, Hue L, Pipeleers D, Van de Casteele M (2003). AMP-activated protein kinase can induce apoptosis of insulin-producing MIN6 cells through stimulation of c-Jun-N-terminal kinase. J Mol Endocrinol.

[B11] Meisse D, Van de Casteele M, Beauloye C, Hainault I, Kefas BA, Rider MH, Foufelle F, Hue L (2002). Sustained activation of AMP-activated protein kinase induces c-Jun N-terminal kinase activation and apoptosis in liver cells. FEBS Lett.

[B12] Dagon Y, Avraham Y, Berry EM (2006). AMPK activation regulates apoptosis, adipogenesis, and lipolysis by eIF2alpha in adipocytes. Biochem Biophys Res Commun.

[B13] Pui CH, Evans WE (1998). Acute lymphoblastic leukemia. N Engl J Med.

[B14] Pui CH, Evans WE (2006). Treatment of acute lymphoblastic leukemia. N Engl J Med.

[B15] Gokbuget N, Hoelzer D (2006). Treatment of adult acute lymphoblastic leukemia. Hematology Am Soc Hematol Educ Program.

[B16] Rattan R, Giri S, Singh AK, Singh I (2005). 5-Aminoimidazole-4-carboxamide-1-beta-D-ribofuranoside inhibits cancer cell proliferation in vitro and in vivo via AMP-activated protein kinase. J Biol Chem.

[B17] Xiang X, Saha AK, Wen R, Ruderman NB, Luo Z (2004). AMP-activated protein kinase activators can inhibit the growth of prostate cancer cells by multiple mechanisms. Biochem Biophys Res Commun.

[B18] Woods A, Johnstone SR, Dickerson K, Leiper FC, Fryer LG, Neumann D, Schlattner U, Wallimann T, Carlson M, Carling D (2003). LKB1 is the upstream kinase in the AMP-activated protein kinase cascade. Curr Biol.

[B19] Astrinidis A, Henske EP (2005). Tuberous sclerosis complex: linking growth and energy signaling pathways with human disease. Oncogene.

[B20] Hemminki A, Markie D, Tomlinson I, Avizienyte E, Roth S, Loukola A, Bignell G, Warren W, Aminoff M, Hoglund P, Jarvinen H, Kristo P, Pelin K, Ridanpaa M, Salovaara R, Toro T, Bodmer W, Olschwang S, Olsen AS, Stratton MR, de la Chapelle A, Aaltonen LA (1998). A serine/threonine kinase gene defective in Peutz-Jeghers syndrome. Nature.

[B21] Jenne DE, Reimann H, Nezu J, Friedel W, Loff S, Jeschke R, Muller O, Back W, Zimmer M (1998). Peutz-Jeghers syndrome is caused by mutations in a novel serine threonine kinase. Nat Genet.

[B22] Nakanishi C, Yamaguchi T, Iijima T, Saji S, Toi M, Mori T, Miyaki M (2004). Germline mutation of the LKB1/STK11 gene with loss of the normal allele in an aggressive breast cancer of Peutz-Jeghers syndrome. Oncology.

[B23] Forcet C, Etienne-Manneville S, Gaude H, Fournier L, Debilly S, Salmi M, Baas A, Olschwang S, Clevers H, Billaud M (2005). Functional analysis of Peutz-Jeghers mutations reveals that the LKB1 C-terminal region exerts a crucial role in regulating both the AMPK pathway and the cell polarity. Hum Mol Genet.

[B24] Inoki K, Zhu T, Guan KL (2003). TSC2 mediates cellular energy response to control cell growth and survival. Cell.

[B25] Li Y, Corradetti MN, Inoki K, Guan KL (2004). TSC2: filling the GAP in the mTOR signaling pathway. Trends Biochem Sci.

[B26] Stapleton D, Woollatt E, Mitchelhill KI, Nicholl JK, Fernandez CS, Michell BJ, Witters LA, Power DA, Sutherland GR, Kemp BE (1997). AMP-activated protein kinase isoenzyme family: subunit structure and chromosomal location. FEBS Lett.

[B27] Shaw RJ, Lamia KA, Vasquez D, Koo SH, Bardeesy N, Depinho RA, Montminy M, Cantley LC (2005). The kinase LKB1 mediates glucose homeostasis in liver and therapeutic effects of metformin. Science.

[B28] Woods A, Vertommen D, Neumann D, Turk R, Bayliss J, Schlattner U, Wallimann T, Carling D, Rider MH (2003). Identification of phosphorylation sites in AMP-activated protein kinase (AMPK) for upstream AMPK kinases and study of their roles by site-directed mutagenesis. J Biol Chem.

[B29] Hunger SP, Sun T, Boswell AF, Carroll AJ, McGavran L (1997). Hyperdiploidy and E2A-PBX1 fusion in an adult with t(1;19)+ acute lymphoblastic leukemia: case report and review of the literature. Genes Chromosomes Cancer.

[B30] Borkhardt A, Harbott J, Lampert F (1999). Biology and clinical significance of the TEL/AML1 rearrangement. Curr Opin Pediatr.

[B31] Greaves M (2002). Childhood leukaemia. Bmj.

[B32] Pui CH, Chessells JM, Camitta B, Baruchel A, Biondi A, Boyett JM, Carroll A, Eden OB, Evans WE, Gadner H, Harbott J, Harms DO, Harrison CJ, Harrison PL, Heerema N, Janka-Schaub G, Kamps W, Masera G, Pullen J, Raimondi SC, Richards S, Riehm H, Sallan S, Sather H, Shuster J, Silverman LB, Valsecchi MG, Vilmer E, Zhou Y, Gaynon PS, Schrappe M (2003). Clinical heterogeneity in childhood acute lymphoblastic leukemia with 11q23 rearrangements. Leukemia.

[B33] Leclerc GJ, Leclerc GM, Kinser TT, Barredo JC (2006). Analysis of folylpoly-gamma-glutamate synthetase gene expression in human B-precursor ALL and T-lineage ALL cells. BMC Cancer.

[B34] Parkinson FE, Geiger JD (1996). Effects of iodotubercidin on adenosine kinase activity and nucleoside transport in DDT1 MF-2 smooth muscle cells. J Pharmacol Exp Ther.

[B35] Massague J (2004). G1 cell-cycle control and cancer. Nature.

[B36] Wang B, Matsuoka S, Carpenter PB, Elledge SJ (2002). 53BP1, a mediator of the DNA damage checkpoint. Science.

[B37] Schafer ZT, Kornbluth S (2006). The apoptosome: physiological, developmental, and pathological modes of regulation. Dev Cell.

[B38] Brancho D, Tanaka N, Jaeschke A, Ventura JJ, Kelkar N, Tanaka Y, Kyuuma M, Takeshita T, Flavell RA, Davis RJ (2003). Mechanism of p38 MAP kinase activation in vivo. Genes Dev.

[B39] Ge B, Gram H, Di Padova F, Huang B, New L, Ulevitch RJ, Luo Y, Han J (2002). MAPKK-independent activation of p38alpha mediated by TAB1-dependent autophosphorylation of p38alpha. Science.

[B40] Lisnock J, Tebben A, Frantz B, O'Neill EA, Croft G, O'Keefe SJ, Li B, Hacker C, de Laszlo S, Smith A, Libby B, Liverton N, Hermes J, LoGrasso P (1999). Molecular basis for p38 protein kinase inhibitor specificity. Biochemistry.

[B41] Davies SP, Reddy H, Caivano M, Cohen P (2000). Specificity and mechanism of action of some commonly used protein kinase inhibitors. Biochem J.

[B42] Luo J, Manning BD, Cantley LC (2003). Targeting the PI3K-Akt pathway in human cancer: rationale and promise. Cancer Cell.

[B43] Vivanco I, Sawyers CL (2002). The phosphatidylinositol 3-Kinase AKT pathway in human cancer. Nat Rev Cancer.

[B44] Stefanelli C, Stanic I, Bonavita F, Flamigni F, Pignatti C, Guarnieri C, Caldarera CM (1998). Inhibition of glucocorticoid-induced apoptosis with 5-aminoimidazole-4-carboxamide ribonucleoside, a cell-permeable activator of AMP-activated protein kinase. Biochem Biophys Res Commun.

[B45] Kato K, Ogura T, Kishimoto A, Minegishi Y, Nakajima N, Miyazaki M, Esumi H (2002). Critical roles of AMP-activated protein kinase in constitutive tolerance of cancer cells to nutrient deprivation and tumor formation. Oncogene.

[B46] Bolster DR, Crozier SJ, Kimball SR, Jefferson LS (2002). AMP-activated protein kinase suppresses protein synthesis in rat skeletal muscle through down-regulated mammalian target of rapamycin (mTOR) signaling. J Biol Chem.

[B47] Swinnen JV, Beckers A, Brusselmans K, Organe S, Segers J, Timmermans L, Vanderhoydonc F, Deboel L, Derua R, Waelkens E, De Schrijver E, Van de Sande T, Noel A, Foufelle F, Verhoeven G (2005). Mimicry of a cellular low energy status blocks tumor cell anabolism and suppresses the malignant phenotype. Cancer Res.

[B48] Shaw RJ, Bardeesy N, Manning BD, Lopez L, Kosmatka M, DePinho RA, Cantley LC (2004). The LKB1 tumor suppressor negatively regulates mTOR signaling. Cancer Cell.

[B49] Imamura K, Ogura T, Kishimoto A, Kaminishi M, Esumi H (2001). Cell cycle regulation via p53 phosphorylation by a 5'-AMP activated protein kinase activator, 5-aminoimidazole- 4-carboxamide-1-beta-D-ribofuranoside, in a human hepatocellular carcinoma cell line. Biochem Biophys Res Commun.

[B50] Igata M, Motoshima H, Tsuruzoe K, Kojima K, Matsumura T, Kondo T, Taguchi T, Nakamaru K, Yano M, Kukidome D, Matsumoto K, Toyonaga T, Asano T, Nishikawa T, Araki E (2005). Adenosine monophosphate-activated protein kinase suppresses vascular smooth muscle cell proliferation through the inhibition of cell cycle progression. Circ Res.

[B51] Jones RG, Plas DR, Kubek S, Buzzai M, Mu J, Xu Y, Birnbaum MJ, Thompson CB (2005). AMP-activated protein kinase induces a p53-dependent metabolic checkpoint. Mol Cell.

[B52] Jimenez AI, Fernandez P, Dominguez O, Dopazo A, Sanchez-Cespedes M (2003). Growth and molecular profile of lung cancer cells expressing ectopic LKB1: down-regulation of the phosphatidylinositol 3'-phosphate kinase/PTEN pathway. Cancer Res.

[B53] Rosner M, Hengstschlager M (2004). Tuberin binds p27 and negatively regulates its interaction with the SCF component Skp2. J Biol Chem.

[B54] Miloloza A, Rosner M, Nellist M, Halley D, Bernaschek G, Hengstschlager M (2000). The TSC1 gene product, hamartin, negatively regulates cell proliferation. Hum Mol Genet.

[B55] Pasumarthi KB, Nakajima H, Nakajima HO, Jing S, Field LJ (2000). Enhanced cardiomyocyte DNA synthesis during myocardial hypertrophy in mice expressing a modified TSC2 transgene. Circ Res.

[B56] Soucek T, Yeung RS, Hengstschlager M (1998). Inactivation of the cyclin-dependent kinase inhibitor p27 upon loss of the tuberous sclerosis complex gene-2. Proc Natl Acad Sci U S A.

[B57] Wullschleger S, Loewith R, Hall MN (2006). TOR signaling in growth and metabolism. Cell.

